# Development and Evaluation of a Reconfigurable 3D LiDAR Sensor for Improved Perception in Autonomous Systems

**DOI:** 10.3390/s26154829

**Published:** 2026-07-30

**Authors:** Bagathi Nithul, Kotaprolu Sai Smaran, Prabakaran Veerajagadheswar, Megalingam Rajesh Kannan, Rajesh Elara Mohan

**Affiliations:** 1 Engineering Product Development Pillar, Singapore University of Technology and Design, Singapore 487372, Singapore; saismaran_kotaprolu@alumni.sutd.edu.sg (K.S.S.); prabakaran@sutd.edu.sg (P.V.); rajeshelara@sutd.edu.sg (R.E.M.); 2 Department of Electronics and Communication Engineering, Amrita Vishwa Vidyapeetham, Amritapuri 690525, India; rajeshm@am.amrita.edu

**Keywords:** 3D CS LiDAR, angular resolution, customizable, reconfigurable

## Abstract

Light detection and ranging (LiDAR) is widely used in robotics, autonomous vehicles, remote sensing, and object tracking, and a large number of commercial 3D LiDAR sensors with diverse specifications are now available. However, these sensors are typically sold with fixed specifications: their measuring range, field of view (FoV), angular resolution, number of scan points, and number of scan layers are all determined at the point of manufacture and cannot be adapted to the application. This rigidity forces the surrounding platform to absorb the cost of excess data, higher computation, larger post-processing storage, and false feature detections even when only a narrow region of interest is needed. To address these limitations, this paper presents 3D Customizable LiDAR (3D CS LiDAR), a novel reconfigurable 3D LiDAR architecture that exposes sensor specifications as run-time parameters. The paper describes the mechanical, electrical, and software subsystems of the developed sensor and evaluates its object-detection performance against a commercial 32-channel LiDAR under two experimental setups. Within the scope of the indoor evaluation reported here (1–3 m range, three geometric targets), the proposed reconfigurable 3D LiDAR provides denser on-target sampling and lower false negative rates than the commercial reference sensor in every tested configuration, indicating its potential for close-range indoor perception tasks such as robotic inspection and short-range obstacle detection. This work contributes to LiDAR technology by introducing a run-time-reconfigurable approach that addresses the specification rigidity of existing sensors and outlines the initial progress towards a full-fledged reconfigurable 3D LiDAR system, with outdoor operation and long-range characterisation identified as future work.

## 1. Introduction

Advances in robotics and autonomous vehicles over the past decade have made perception—the ability of an autonomous system to sense, interpret, and act upon its physical surroundings—a central engineering concern. An autonomous platform must build and maintain a spatial model of its environment sufficiently accurate to plan safe motion around obstacles and to complete task-specific interactions. A range of sensing modalities is used for this purpose, including cameras, ultrasonic ranging, sonar, and Light Detection and Ranging (LiDAR). LiDAR is a particularly common choice because it directly returns range to the sensed surface, is largely invariant to visible light illumination, and produces a spatial representation that can be consumed directly by mapping and obstacle avoidance algorithms.

LiDAR can detect small objects and produce a precise layout of the space in which it operates [1]. The sensor’s ability to transmit laser pulses and receive their reflections within nanoseconds allows broad areas to be scanned rapidly while returning a large volume of range data. LiDARs have been employed across a broad range of applications for the past several decades. The technology is not new: as early as 1971, NASA used a LiDAR to map the surface of the moon [2]. Present-day applications include aerial topographic mapping and, in specialised marine variants, mapping of sub-sea surfaces.

In contemporary robotics, LiDARs are used primarily for environment mapping, robot localisation, and obstacle detection. Several studies have demonstrated LiDAR-based path planning and navigation in unknown environments [3,4,5], and others have documented the sensor’s role in the development of autonomous vehicles [6,7]. A survey by Roriz et al. [8] concludes that LiDAR is well suited to translating the physical world into 3D digital representations in real time for automotive use. McLeod et al. [9] further show that LiDAR outperforms conventional acoustic methods for the mapping of sub-sea infrastructure. Collectively, these works establish LiDAR as a mature primary modality for spatial perception in autonomous systems. Over the last decade, the introduction of 3D LiDAR has extended this capability from 2D range mapping to full three-dimensional environmental sensing.

Commercial 3D LiDAR classes are generally distinguished by three specification axes: vertical field of view (FoV), number of scanning channels, and maximum sensing range. Lambert et al. [10] benchmarked ten commercial 3D LiDAR models—including the Velodyne VLP-16 and HDL-32—across FoV, laser intensity, scan pattern, price, and channel count, and showed that no single model is optimal across all deployment classes. In practice, users select a sensor to match an assumed operating envelope: a 16-channel LiDAR is typical for semi-outdoor mobile robots, a 32-channel unit with ∼100 m range is common for aerial platforms, and 64- or 128-channel units are reserved for outdoor autonomous vehicles.

While this class-based selection is workable, it exposes a structural limitation: once purchased, the sensor’s specifications are fixed. The user cannot adapt channel count, angular resolution, FoV, or range at run-time. This rigidity produces three practical problems for perception engineers. First, when a target of interest falls outside the fixed vertical FoV—for example, an object placed on an elevated surface relative to a mobile robot—it is under-sampled or missed entirely, regardless of how close it is. Second, when a target is small or distant relative to the sensor’s angular resolution, it is represented by too few laser returns to support reliable feature extraction, again independently of the sensor’s nominal specifications. Third, when the sensor is deployed in an environment much wider than the region of interest, it produces excess points that inflate downstream computational cost, memory footprint, and the probability of false feature detection.

A LiDAR that permitted the user to specify FoV, number of output layers, and range at run-time would address all three problems simultaneously: the sensor could concentrate its sampling on the region occupied by the target, reduce data volume outside that region, and thereby reduce the computational burden on the perception stack. To the best of our knowledge, no commercially available 3D LiDAR provides this level of run-time reconfigurability of the output point cloud specifications. This gap motivates the present work.

In this paper, we propose a novel reconfigurable 3D LiDAR, namely, 3D CS LiDAR, which has the flexibility to adjust the number of layers needed, the field of view, and the range for a particular application. The main contributions of this paper are as follows:

- Design and development of a reconfigurable 3D LiDAR system that can adjust its number of channels, the field of view, and the range.
- Setting up an experimental scenario wherein the developed system can be evaluated methodically.
- Evaluating the developed sensor in terms of object feature detection through systematically benchmarking it with a commercially available fixed 3D LiDAR.

This paper detailed all these aspects and the translation of the theoretical design into a physical system.

*Scientific novelty.* We acknowledge that the general mechanical approach of steering a single ToF ranger with a pan-tilt actuator pair is not new; a variety of hobbyist and research systems have used similar arrangements to generate low-cost 3D point clouds. The contribution of this work is therefore not the pan-tilt geometry itself, but the run-time reconfigurable sensing envelope that this geometry is used to expose. Concretely, three parameters that are hard-coded into conventional 3D LiDARs at manufacture—the angular field of view (both horizontal and vertical), the number of output layers, and the sensing range—are exposed as run-time inputs of the perception pipeline, without any hardware change between successive scans. To the best of our knowledge, this run-time reconfigurability has not been demonstrated for a 3D LiDAR: prior variants either fix the resolution and vary only the mounting geometry (e.g., [11], multiple 2D LiDARs on a rotating plane) or improve post-processing of a fixed specification sensor’s output (e.g., [12], Ripley’s K on a VLP-16). The novelty we claim is therefore twofold: (i) a compact mechatronic architecture in which a single ToF ranger is steered by two orthogonal actuators whose step sizes are computed at run-time from user-supplied FoV and layer count parameters (Equations (2) and (3)); and (ii) a software–hardware pipeline that propagates these user parameters through a ROS control node to the actuators and back to the point cloud generator, closing the loop between application intent and sensor sampling density in real time. The empirical consequence—confirmed quantitatively in Section 4—is that the same physical sensor can be re-tasked from a wide-area survey configuration to a narrow, high-density inspection configuration without hardware changes.

*Scope of reconfiguration in the present study.* We clarify that reconfigurability, as demonstrated in this paper, is the ability to specify a new set of scan parameters (FoV bounds, layer count, and range) at the beginning of each scan—that is, between-scan reconfiguration. Mid-scan re-parameterisation, in which the sensor changes its sampling policy during a single sweep in response to intermediate observations, is a natural but distinct extension that would require an interruptible actuator control loop and an online decision policy; this is identified as future work (Item 4 of the Conclusion, in conjunction with the automatic reconfiguration item).

### Related Works

Related work on LiDAR falls into four broad categories: environmental mapping and feature extraction, object detection, SLAM and navigation, and LiDAR hardware design. We summarise each in turn and then position the present work with respect to prior reconfigurable variants.

*Mapping and feature extraction.* Early studies focused on using LiDAR point clouds to construct 3D representations of physical environments. Reference [13] presented a cost-efficient 3D visualisation pipeline for indoor spaces. Mayura and Veni [14] proposed a classification method that distinguishes buildings from vegetation directly from LiDAR data. These works establish the point cloud as the primary intermediate representation of interest for downstream perception.

*Object detection.* A second body of literature applies LiDAR to the direct detection of discrete objects in the sensed environment. Catapang and Ramos [15] demonstrated a low-cost 360° obstacle detection system using the LiDAR-Lite v1 sensor. Sahba et al. [16] show that increasing the number of LiDAR sweeps improves the performance of 3D object detectors. Urmila and Megalingam [17] reported a LiDAR-based traffic scene perception model, achieving 90% detection accuracy across pedestrians, vehicles, and traffic signals.

*SLAM and navigation.* A third body of work integrates LiDAR into simultaneous localisation and mapping (SLAM) pipelines. Shin et al. [18] fuse LiDAR with camera and IMU data to obtain pose and odometry estimates. Ocando et al. [19] demonstrate autonomous 3D reconstruction from a single 2D LiDAR. Chan et al. [20] present a LiDAR-based ROS/Gazebo simulation framework for 3D SLAM in featureless commercial and industrial interiors. Xue et al. [21] propose LeGO-LOAM-SC, an improved SLAM algorithm fusing LeGO-LOAM with Scan Context for underground environments. Megalingam et al. [22,23,24] evaluate mobile-robot SLAM adaptability under both static and dynamic conditions and demonstrate GUI-driven indoor navigation stacks.

*LiDAR hardware and reconfigurability.* A fourth body of work—most directly relevant to the present paper—proposes design-level modifications to the LiDAR itself in pursuit of higher performance, better output resolution, or reduced cost. Pena Queralta et al. [11] construct a 3D LiDAR by mounting multiple 2D LiDARs on a rotating plane, with FPGA-side processing to produce a 3D image. Flottmann et al. [25] address the energy and real-time constraints of SLAM in autonomous robots. Morales et al. [12] target the low vertical resolution of 3D LiDARs by applying Ripley’s K function to point clouds acquired from a rotating VLP-16 platform. Schulte-Tigges et al. [26] benchmark six commercial 3D LiDARs (including Livox Horizon and RoboSense M1) across static and dynamic scenarios. Li et al. [27] review conventional solid-state LiDARs and emerging nanophotonics-based variants. Elhousni and Huang [28] focus on LiDAR-based localisation for autonomous driving. Complementary to these LiDAR-side studies, reconfigurable robot platforms have been explored more broadly: Prabakaran et al. [29] present a Tetris-inspired reconfigurable cleaning robot, and follow-on studies [30,31] develop path-planning and A*-based control algorithms for such platforms. Beyond the LiDAR modality itself, related work on the restoration of degraded sensor data in adjacent imaging modalities—for example, diffusion-based dehazing of remote-sensing imagery [32] and Bayesian variational restoration of nighttime images [33]—suggests directions from which analogous point cloud enhancement techniques may be adapted; we return to this line of work in the future work discussion of Section 5.

Positioning: existing LiDAR hardware works modify the mounting geometry (multiple 2D LiDARs on a rotating plane, [11]), the on-board processing pipeline (FPGA-accelerated SLAM, [25]), or the post-processing of a fixed specification sensor’s output (Ripley’s K on a VLP-16, [12]). None of these expose the sensor’s specifications—FoV, number of layers, and range—as run-time inputs to the perception pipeline. This is the gap addressed by the present work and is elaborated in the Scientific Novelty statement above.

## 2. System Architecture

Two hardware prototypes of the proposed sensor were developed: Model-A and Model-B, where Model-B is an incremental upgrade of Model-A that replaces the elevation actuator to raise the achievable vertical resolution.

### 2.1. Model-A

**Figure 1:**
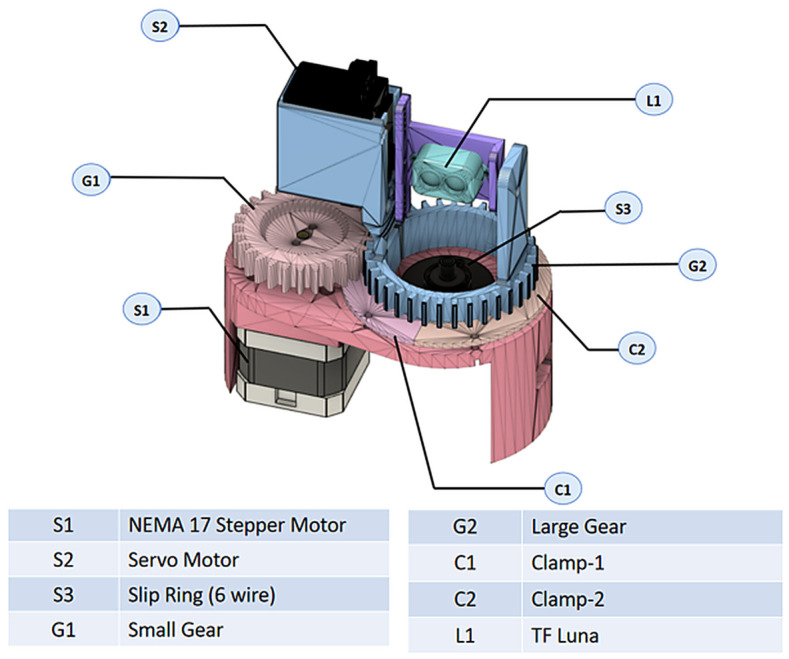
3D CS LiDAR—Model-A.

Commercial 3D LiDARs are typically constructed from an array of time-of-flight (ToF) sensors arranged in a fixed geometric pattern that determines the sensor’s field of view and layer count at manufacture. The proposed 3D CS LiDAR departs from this convention by using a single ToF ranger together with a pair of orthogonal actuators, so that the sensor’s effective FoV and layer count can be varied at run-time under software control. Model-A is the first prototype instantiation of this architecture and is shown in Figure 1.

#### 2.1.1. Design

As shown in Figure 1, the LiDAR comprises a mounting base on which two adjacent gears—a small drive gear (Figure 1—G1) with 28 teeth and a large driven gear (Figure 1—G2) with 35 teeth—are mounted. The gear pair is selected on the basis of ratio, size, and tooth engagement, giving an overall reduction of 28:35. The large gear is locked to the base by two C-clamps (Figure 1—C1 and C2) to prevent wobble during rotation. A TF Luna ToF ranger (Benewake Co., Ltd., Beijing, China) (Figure 1—L1) with an operating range of 8 m and a single-beam field of view of 2° is mounted on the large gear and provides range measurements to the target. An Arduino UNO (Arduino S.r.l., Monza, Italy) acts as the microcontroller. A NEMA-17 bipolar stepper motor (StepperOnline, Nanjing, China) (Figure 1—S1) with a holding torque of 59 N·cm, driven by a TB6600 motor driver (Toshiba Corporation, Tokyo, Japan), provides 360° azimuthal (horizontal) motion. A Towerpro MG995 servo motor (Tower Pro Pte. Ltd., Shenzhen, China) (Figure 1—S2) operating at 5 V with a stall torque of 10 kg·cm provides elevation (vertical) motion. A 6-wire slip ring (Senring Electronics Co., Ltd., Shenzhen, China) (Figure 1—S3) prevents cable winding during continuous azimuthal rotation and thereby ensures smooth, uninterrupted operation.

#### 2.1.2. Working Principle of Model-A

**Figure 2:**
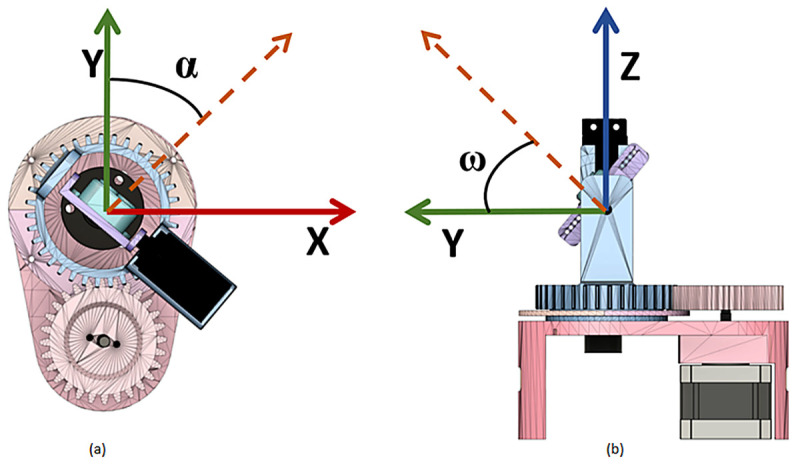
Coordinate system developed towards designing the 3D CS LiDAR. (**a**) Top view of the coordinate system (**b**) Side view of the coordinate system.

Figure 2a,b show the top and side views of the LiDAR coordinate frame, respectively. The frame is spherical, consistent with the fact that the ToF sensor rotates about a fixed origin at a variable radius of measurement *r*. The azimuthal angle α is defined as the angle between the target point’s projection on the XY plane and the *Y* axis; the elevation angle ω is the angle between the target point and the *Y* axis; and the range *r* is the distance from the origin to the target point. The point cloud is generated by transforming each measured spherical triple (α,ω,r) into Cartesian coordinates (X,Y,Z).

Formally, at each sampling instant *k* the sensor returns a triple $\left(\alpha_{k},\omega_{k},r_{k}\right)$, where $\alpha_{k}\in \left[0,2\pi \right)$ is the azimuth commanded by the horizontal stepper, $\omega_{k}\in \left[\alpha_{min},\alpha_{max}\right]$ is the elevation commanded by the vertical actuator, and $r_{k}\in \left[r_{min},r_{max}\right]$ is the range returned by the TF Luna ToF sensor. The spherical-to-Cartesian mapping applied by the ROS node is

(1) $$\left[\begin{array}{c} x_{k}\\ y_{k}\\ z_{k} \end{array}\right]=r_{k}\left[\begin{array}{c} \sin\omega_{k}\cos\alpha_{k}\\ \sin\omega_{k}\sin\alpha_{k}\\ \cos\omega_{k} \end{array}\right],$$

so that the full output point cloud is $P={\left\{\left(x_{k},y_{k},z_{k}\right)\right\}}_{k=1}^{K}$, with $K=N_{\alpha }\cdot n$, where $N_{\alpha }$ is the number of azimuthal samples per full horizontal rotation and *n* is the user-specified number of vertical layers. The reconfigurability of the sensor is captured by the fact that $\alpha_{min}$, $\alpha_{max}$, *n*, $r_{min}$, and $r_{max}$ are all supplied at run-time rather than being fixed at manufacture. The angular sampling density on the target is therefore a controllable variable of the perception pipeline, not a hardware constant.

Operationally, the motion of the 3D LiDAR can be viewed as a vertical stack of 2D azimuthal scans: for each full 360° azimuthal sweep of the stepper, the elevation actuator increments the TF Luna’s pitch by a step angle derived from the user parameters, and the sequence repeats until the full elevation span has been covered. The azimuth angle is measured from the stepper (whose axis of rotation is the sensor’s *Z* axis), the elevation angle is measured from the vertical actuator, and the range is provided by the ToF sensor.

The Arduino UNO coordinates the actuators and the sensor. When the user supplies the maximum elevation angle $\alpha_{max}$, the minimum elevation angle $\alpha_{min}$, and the desired number of output layers *n*, the microcontroller computes the vertical step size (i.e., the effective vertical resolution) as the elevation span divided by *n*, and applies it after every completed azimuthal sweep of the stepper. The number of output layers is therefore controlled directly by this step size, which is given by Equation (2).

(2) $$\Delta \alpha =\frac{\alpha_{max}-\alpha_{min}}{n}$$

where Δα is the step angle of the servo motor (i.e., the vertical angular resolution), $\alpha_{max}$ is the user-specified maximum elevation angle, $\alpha_{min}$ is the user-specified minimum elevation angle, and *n* is the desired number of output scan layers.

**Figure 3:**
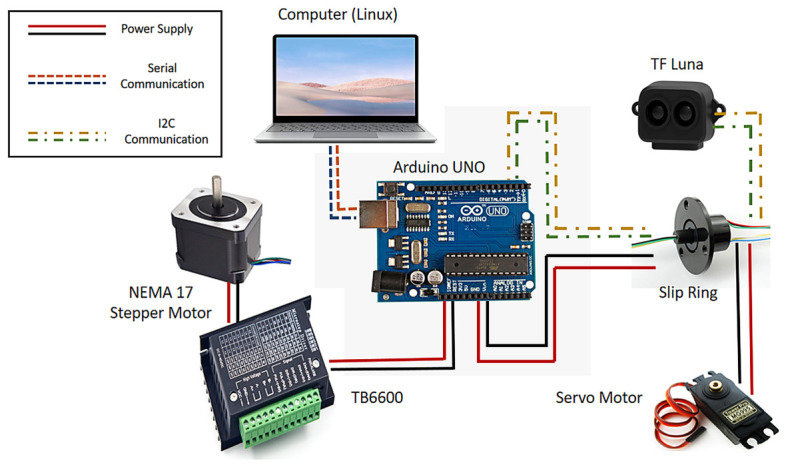
System architecture for 3D LiDAR Model-A.

Although the outcomes of the tests performed with Model-A were satisfactory, the vertical resolution of the output point cloud was limited by the servo motor, whose smallest achievable step angle is $1^{\circ }$. For example, if the user specifies a minimum elevation angle of $50^{\circ }$ and a maximum of $100^{\circ }$, the elevation span is $50^{\circ }$. Substituting into Equation (2) with $\Delta \alpha \geq 1^{\circ }$, the maximum number of output layers is bounded by $n\leq 50^{\circ }/1^{\circ }=50$ layers. The resolution therefore cannot exceed $1^{\circ }$ per layer. Figure 3 shows the overall architecture of Model-A 3D CS LiDAR.

This resolution ceiling motivates the redesign presented in Section 2.2. Model-B replaces the servo with a fine-step stepper motor, raising the achievable layer count by more than an order of magnitude.

*Effect of vertical-actuator characteristics on point cloud quality.* The choice of vertical actuator directly and quantifiably determines two properties of the output point cloud. First, the minimum achievable angular step of the actuator, $\theta_{min}$, is a hard upper bound on the achievable vertical resolution: Equation (3) shows that the maximum number of layers is $m=\left(\alpha_{max}-\alpha_{min}\right)/\theta_{min}$, so a smaller $\theta_{min}$ increases the achievable layer density linearly. Replacing the Model-A servo ($\theta_{min}=1^{\circ }$) with the Model-B 28BYJ-48 stepper ($\theta_{min}=0.18^{\circ }$) therefore raises the ceiling on layer count by a factor of ∼5.5 for the same elevation span. Second, actuator step-position repeatability sets a lower bound on the per-point angular noise: any deviation from the commanded step angle, either due to backlash or micro-step positioning error, is directly injected into the elevation coordinate $\omega_{k}$ of Equation (1), producing a vertical positional error of order $r_{k}\sin\left(\Delta \omega_{err}\right)$ on the target. The stepper-based Model-B, having no PWM-driven analogue position hold, is expected to be less susceptible to this class of error than the servo-based Model-A—a hypothesis consistent with the higher on-target layer counts reported for Model-B in Section 4.1.4.

### 2.2. Model-B

**Figure 4:**
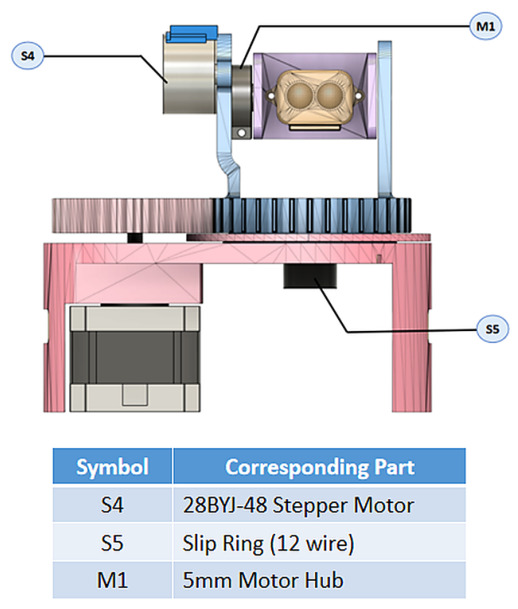
Mechanical CAD model of Model-B.S4 is the stepper motor used to exert vertical motion to our LiDAR, S5 is the 12-wire slip ring used to avoid any sort of winding up of wires which enables smoother rotation.

**Figure 5:**
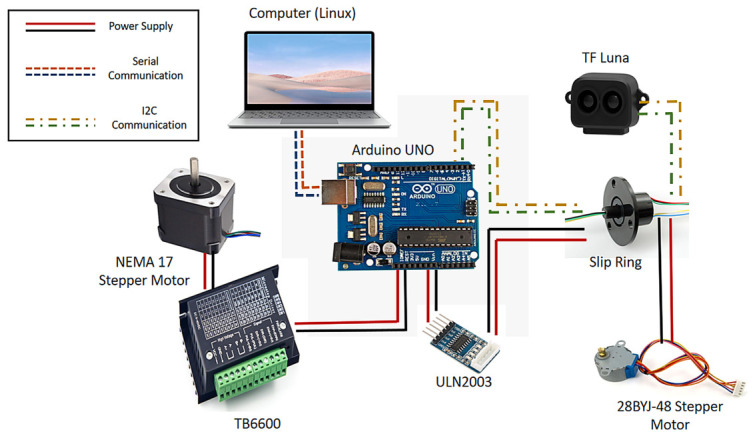
System architecture for 3D LiDAR Model-B.

Model-B retains the mechanical architecture of Model-A with a single change: the vertical actuator is replaced by a 28BYJ-48 stepper motor (Kiatronics, Shenzhen, China) (Figure 4—S4) driven through a ULN2003 driver (Texas Instruments, Dallas, TX, USA).The 28BYJ-48 stepper has a minimum step angle of $0.18^{\circ }$, an order of magnitude finer than the $1^{\circ }$ ceiling of the Model-A servo. As in Model-A, the user retains full run-time control of the minimum and maximum elevation angles and the number of output layers. The corresponding electrical architecture is shown in Figure 5.

The working principle is otherwise identical to Model-A. On receiving the user parameters (minimum elevation angle, maximum elevation angle, number of output layers, and range), the microcontroller computes the required vertical step size for the 28BYJ-48 stepper (Figure 4—S4) using Equation (2). The maximum number of output layers that can be achieved for a given elevation span is bounded by the stepper’s minimum step angle and is given by Equation (3):

(3) $$m=\frac{\alpha_{max}-\alpha_{min}}{\theta_{min}}$$

where *m* is the maximum number of output layers, $\theta_{min}$ is the minimum step angle of the vertical stepper motor (Figure 4—S4), $\alpha_{max}$ is the maximum elevation angle, and $\alpha_{min}$ is the minimum elevation angle. For the 28BYJ-48 stepper used in Model-B, $\theta_{min}=0.18^{\circ }$.

For example, assuming the parameters entered are a maximum elevation angle of $100^{\circ }$ and a minimum elevation angle of $50^{\circ }$, the elevation span is $50^{\circ }$. Applying Equation (3), the maximum number of output layers achievable in the point cloud map is $m=50^{\circ }/0.18^{\circ }\approx 277$ layers. This is a ∼5.5× improvement over the theoretical maximum of Model-A and reflects the primary motivation for the redesign.

### 2.3. Software Design

**Figure 6:**
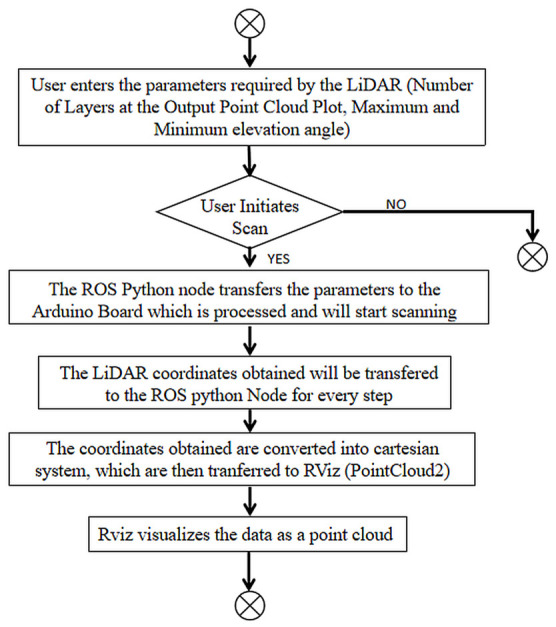
Software design flow of our proposed 3D CS LiDAR.

Both models of the 3D CS LiDAR share the same software pipeline; the corresponding program flow is shown in Figure 6. The system integrates with the Robot Operating System (ROS) for point cloud generation and visualisation. The Arduino UNO is connected to the Linux ROS master over USB using the ROS-serial protocol. On start-up, the user supplies four run-time parameters—the desired sensing range, the minimum and maximum elevation angles, and the number of output layers—which the ROS node forwards to the Arduino before the scan begins. The Arduino then executes the scan according to these parameters and returns to the ROS node the raw LiDAR triples $\left(\alpha_{k},\omega_{k},r_{k}\right)$ obtained from the two actuators and the ToF ranger. A ROS Python (ROS Noetic 1.16, Python 3.8) node transforms these spherical triples into Cartesian coordinates using Equation (1) and publishes the result as a PointCloud2 message that is visualised in RViz.

### 2.4. Technical Specifications

Table 1 summarises the key technical characteristics of both models of the 3D CS LiDAR and the commercial 32-channel LiDAR used as the reference in Section 4. The wavelength, range and ranging precision entries for the proposed sensor are inherited from the TF Luna module’s manufacturer datasheet; the actuator entries reflect the choice of mechanical components; and the reconfigurable-parameter entries reflect the run-time-adjustable envelope of each model.

### 2.5. Physical Considerations for TF Luna ToF Sensing

The TF Luna module used in both Model-A and Model-B is a 940 nm pulsed time-of-flight ranger. Two physical effects place the practical envelope of the sensor. First, at 940 nm the atmospheric transmission window is high in dry indoor conditions but is attenuated by water vapour and by particulate scattering in aerosols, fog, and dust; the range precision quoted in Table 1 is valid under indoor conditions only, and outdoor performance in humid, dusty, or foggy environments would require both a re-characterisation of the sensor and, most likely, a photonics upgrade (e.g., 1550 nm operation for improved atmospheric penetration). Second, the ToF principle requires the sensor to receive a photon that was reflected from a target surface within the electronic ranging window; specular targets at oblique angles, and highly absorbing targets, both reduce the effective returned energy and are known to increase the fringe-related noise mentioned in the list of future work. The experiments reported in Section 4 are therefore conducted in a controlled indoor setting with Lambertian-approximate targets, and the outdoor generalisation of the results is left to future work.

*Scope of noise handling in the current study.* We explicitly note that the present work does not implement or evaluate a dedicated denoising or filtering stage on the returned range values: the TF Luna’s on-module median-filtered range output is passed unmodified through the coordinate transformation of Equation (1) into the visualised point cloud. Two consequences follow. First, the layer count and detection metrics reported in Section 4 reflect the raw sensor behaviour without any denoising benefit, which is a conservative choice—a downstream denoising or point cloud restoration step would only improve the reported results. Second, a full quantitative noise characterisation of the sensor (per-point range error histograms as a function of target angle, distance, and surface reflectance) would require a dedicated calibration campaign against a reference such as a robotic total station, which is beyond the scope of this initial architectural study and is identified as future work. Extension of the pipeline with a LiDAR-appropriate point cloud restoration stage—for instance, statistical outlier removal, radius-based filtering, or a learned denoiser—is a natural next step, since these methods are known to reduce the false feature detection discussed in the Introduction.

### 2.6. Refresh Rate and Data Link Throughput

The scene refresh rate of the 3D CS LiDAR is bounded by two serial processes: (i) the TF Luna’s per-sample acquisition time, and (ii) the mechanical settling of the two actuators between samples. The TF Luna operates at a native sample rate of 100 Hz (i.e., 10 ms per range measurement) as specified in the manufacturer datasheet, and each azimuthal step of the NEMA-17 stepper together with each elevation step of the 28BYJ-48 stepper introduces an additional mechanical dwell that is dominated by the driver step rate and the load inertia. Under the customised scan settings used in Experiment-2 ($N_{\alpha }\approx 360$ azimuthal samples × n=40 layers = 14,400 samples per full-scene refresh), the measured full-scene refresh period is approximately 58 s, i.e., roughly 1/580 of the ∼10 Hz refresh rate typical of a commercial multi-channel LiDAR such as the reference sensor of Table 1. This is an acknowledged and expected limitation of any mechanically actuated single-ToF architecture and is listed as Item 2 of the future work list (major design change required, e.g., by using a multi-ToF fan-out or a MEMS mirror). The comparison with commercial LiDARs in Section 4 is therefore a comparison of *spatial reconfigurability and on-target sampling density at a fixed scan*, not of full-scene refresh throughput.

The data link between the Arduino UNO and the ROS master runs at the ROS-serial default baud of 115,200 bit/s, corresponding to a raw byte throughput of approximately 11.5 kB/s. Each transmitted sample carries three floating-point coordinates plus framing, on the order of 16–20 bytes per point, giving a link-limited maximum of approximately 570–720 points per second. This link throughput is well matched to the mechanical sample rate above (order 50–100 points per second at 10–20 ms per sample), so the ROS-serial link is not the bottleneck of the current pipeline; the mechanical acquisition time dominates. The observed round-trip latency of a single-sample command–acknowledge cycle at 115,200 baud is on the order of 1–2 ms, again well below the mechanical sample budget.

### 2.7. Cost Breakdown

Table 2 lists the component-level cost of the two 3D CS LiDAR prototypes in United States dollars, based on prevailing prices of the individual off-the-shelf modules at the time of build. All prices are indicative and exclude shipping, taxes, and the cost of 3D-printed mechanical parts (fabricated in-house in PLA). The commercial 32-channel LiDAR used as the benchmark is included for reference; commercial multi-channel LiDARs vary in price with model and vendor, and the range shown reflects typical list prices for entry- to mid-tier 32-channel devices.

The bill-of-materials cost of either prototype is therefore approximately 1–2 orders of magnitude lower than the list price of a comparable commercial 32-channel LiDAR, before amortising engineering time or accounting for fabrication of the 3D-printed mechanical structure. This substantiates the “cheaper sensors” remark in the Conclusion, while acknowledging that the comparison is a bill-of-materials comparison, not a full unit economics comparison at production volume.

## 3. Experimental Design

The proposed 3D CS LiDAR was evaluated against a commercial 32-channel 3D LiDAR under two experimental setups. Setup-1 (Section 3.1) evaluates the ability of the sensor to detect individual targets when their horizontal and vertical positions are varied and thereby characterises the sensor’s per-target performance envelope. Setup-2 (Section 3.2) evaluates the sensor in more realistic multi-target scenes with the LiDAR mounted on a mobile robot and tests the ability of the reconfigurability feature to preserve detection performance under variable target elevations and positions.

### 3.1. Experiment Setup-1

**Figure 7:**
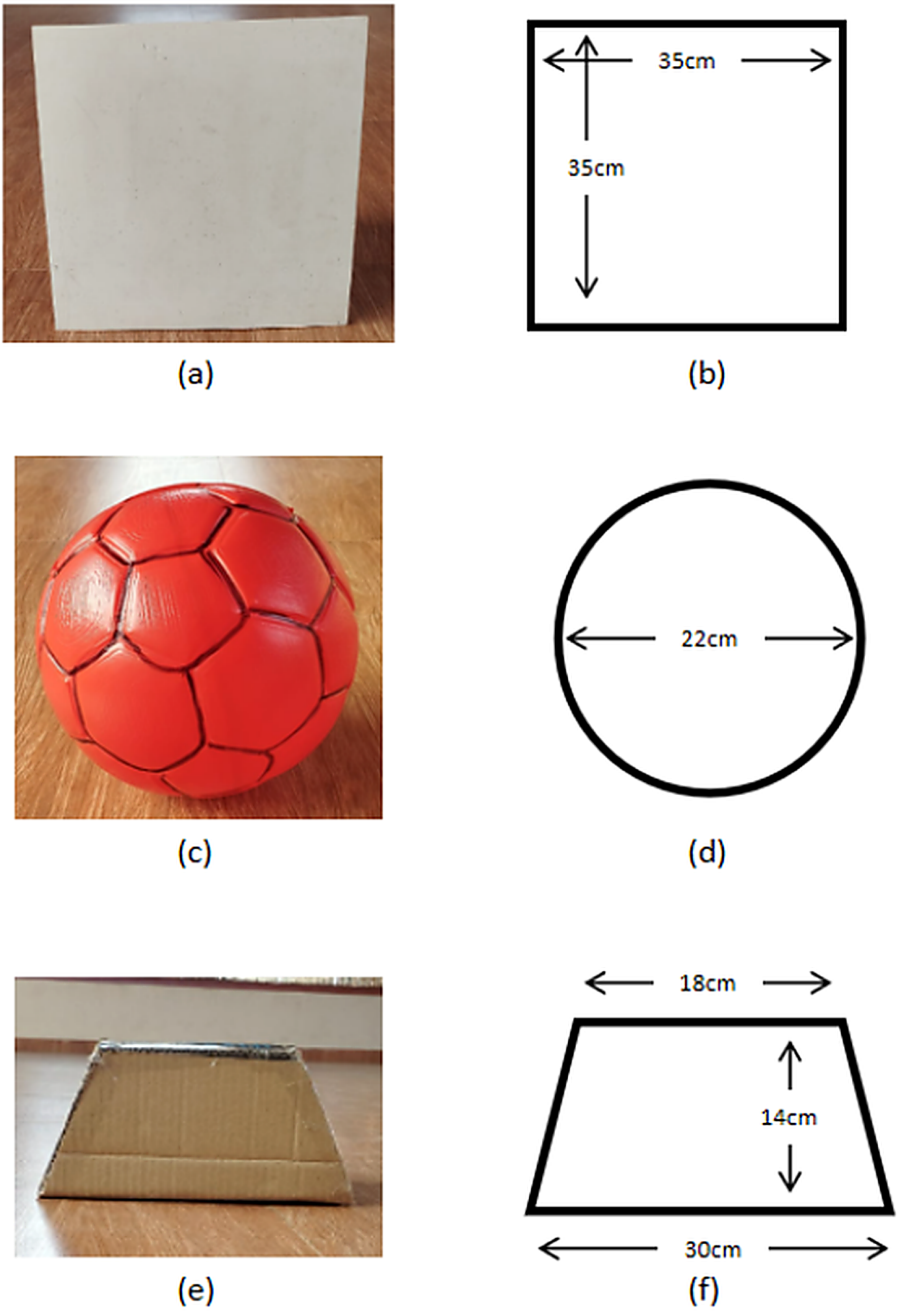
(**a**) Photograph of the plank target; (**b**) schematic of the plank with length and width 35 cm; (**c**) photograph of the ball target; (**d**) schematic of the ball with diameter 22 cm; (**e**) photograph of the trapezium target; (**f**) schematic of the trapezium with base1, base2, and height of 18 cm, 30 cm, and 14 cm, respectively.

**Figure 8:**
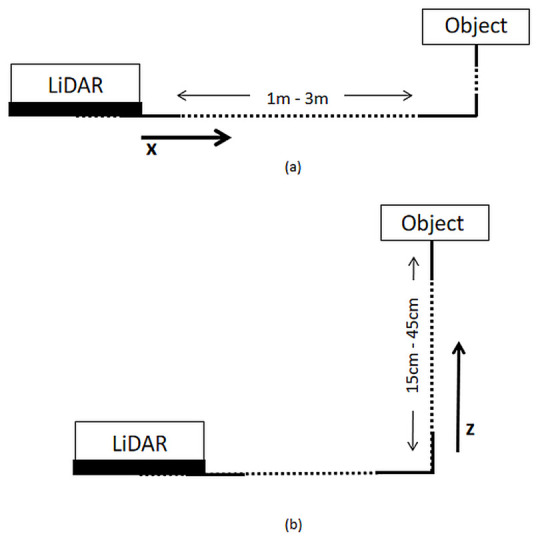
3D CS LiDAR Experiment Design setup-1. (**a**) shows the varying horizontal separation, (**b**) shows the varying vertical separation between the object and the LiDAR.

The customisation feature of the 3D CS LiDAR was tested by scanning three geometric targets across a grid of horizontal distances and vertical elevations. The targets are shown in Figure 7: a plank of length and width 35 cm, a ball of diameter 22 cm, and a trapezoid with base widths of 18 cm and 29 cm and a height of 14 cm. Each target was placed at horizontal distances of 1 m, 2 m, and 3 m from the sensor, and at each distance the vertical elevation was swept through 15 cm, 30 cm, and 45 cm in 15 cm increments (Figure 8). The 3D point cloud produced at every combination was recorded for post-hoc analysis. To assess the improvements introduced by Model-B relative to Model-A, all combinations were scanned with both prototypes; the commercial 32-channel LiDAR was subjected to an identical protocol under the same physical setup to enable a fair comparison.

*Ground-truth measurement.* The horizontal separation between the LiDAR reference plane and each target object was measured using a graduated steel measuring tape with a resolution of ±5 mm, and the vertical elevation of each target above the LiDAR’s mounting plane was measured using the same instrument. Target object centres were aligned to the LiDAR’s optical axis prior to each scan. The overall uncertainty in ground-truth object position is estimated to be within ±10 mm in each axis, which is below the ranging precision of the TF Luna ToF sensor (±2 cm at ≤3 m per the manufacturer datasheet) and is therefore adequate for the layer count and detection metrics reported below.

### 3.2. Experiment Setup-2

In this second series of experiments, the LiDAR was mounted on a mobile robot platform to scan a wider-area environment. The three targets from Setup-1 (plank, ball, and trapezoid) were retained but were placed at a mixture of elevations and horizontal distances from the sensor to evaluate whether the reconfigurability feature can maintain target detection when target height varies across the FoV. All Setup-2 tests were carried out using Model-B, which had already been shown in Setup-1 to be the higher-performing prototype.

Three scenarios were defined, and each was scanned with all comparison sensors. In every scenario the LiDAR was placed on a platform 20 cm above the ground to reflect a realistic deployment height on a mobile robot.

**Figure 9:**
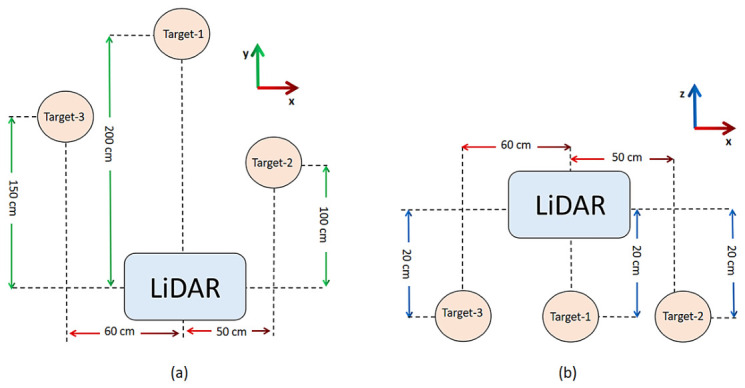
For Scenario-1 of setup-2 (**a**) shows the XY-plane horizontal separation between the LiDAR and the objects, (**b**) shows XZ-plane vertical separation between the LiDAR and the objects.

In *Scenario-1*, all three targets are placed on the ground at varying horizontal distances from the LiDAR, as illustrated in Figure 9. This scenario tests whether the sensor can reliably detect ground-plane obstacles.

**Figure 10:**
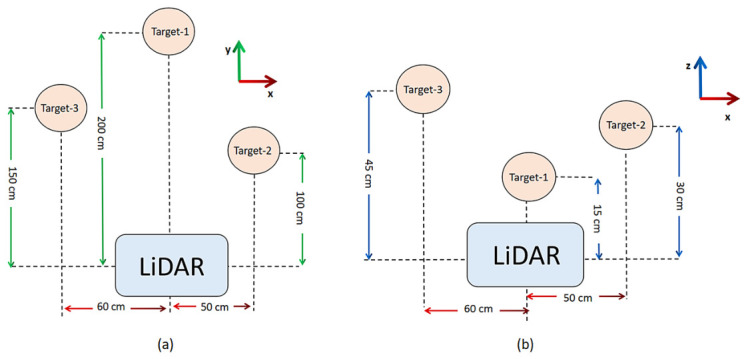
For Scenario-2 of setup-2 (**a**) shows the XY plane-horizontal separation between the LiDAR and the objects, (**b**) shows XZ plane-vertical separation between the LiDAR and the objects.

In *Scenario-2*, some targets are placed on the ground and others at elevation, as illustrated in Figure 10. This mixed configuration probes the reconfigurability benefit directly: a wide fixed FoV is capable of covering all target heights but at correspondingly reduced layer density on each target, whereas the reconfigurable sensor can concentrate its layer budget on the specific vertical band containing the targets.

**Figure 11:**
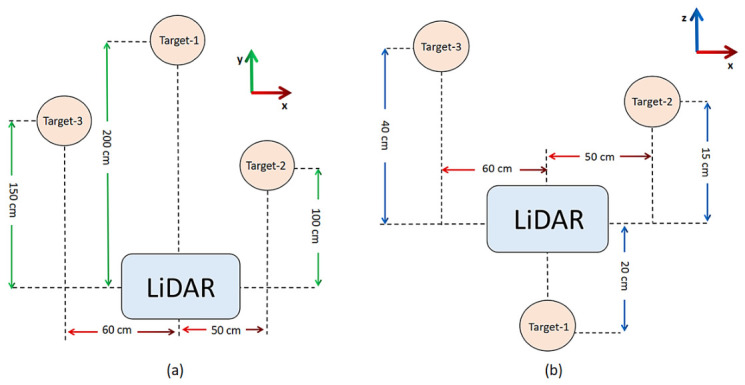
For Scenario-3 of setup-2 (**a**) shows the XY-plane horizontal separation between the LiDAR and the objects, (**b**) shows XZ-plane vertical separation between the LiDAR and the objects.

In *Scenario-3*, all three targets are placed at elevation above the ground, as illustrated in Figure 11, thereby exercising the sensor at large elevation angles and testing performance in the vertical range that a conventional ground-level LiDAR would under-sample.

## 4. Results and Analysis

### 4.1. Results for Experiment-1

As the benchmark, we used a commercially available 32-channel 3D LiDAR with a vertical field of view of 40° ($-25^{\circ }$ to $+15^{\circ }$). The same environments were scanned with this commercial sensor to establish a reference against which the outputs of the proposed 3D CS LiDAR are compared. For clarity of presentation, we organise the results below into three scenarios according to the target object used.

**Figure 12:**
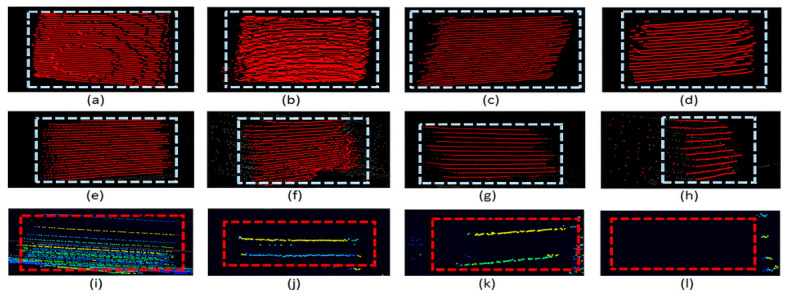
(**a**) Map of plank at 1 m distance without elevation using 3D LiDAR Model-B, (**b**) map of plank at 15 cm height and 1 m distance using 3D LiDAR Model-B, (**c**) map of plank at 30 cm height and 2 m distance using 3D LiDAR Model-B, (**d**) map of plank at 45 cm height and 3 m distance using 3D LiDAR Model-B, (**e**) map of plank at 1 m distance without elevation using 3D LiDAR Model-A, (**f**) map of plank at 15 cm height and 1 m distance using 3D LiDAR Model-A, (**g**) map of plank at 30 cm height and 2 m distance using 3D LiDAR Model-A, (**h**) map of plank at 45 cm height and 3 m distance using 3D LiDAR Model-A, (**i**) map of plank at 1 m distance without elevation using 32-channel LiDAR, (**j**) map of plank at 15 cm height and 1 m distance using 32-channel LiDAR, (**k**) map of plank at 30 cm height and 2 m distance using 32-channel LiDAR, (**l**) map of plank at 45 cm height and 3 m distance using 32-channel LiDAR. Dashed blue box (in panels **a**–**h**) and dashed red box (in panels **i**–**l**) mark the projected extent of the target’s ground-truth bounding box.

**Figure 13:**
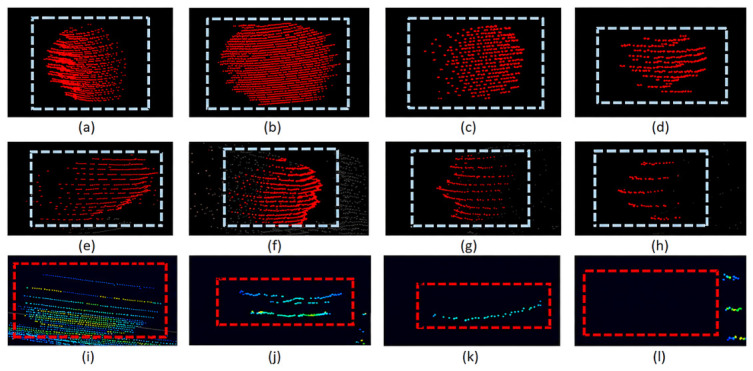
(**a**) map of ball at 1 m distance without elevation using 3D LiDAR Model-B, (**b**) map of ball at 15 cm height and 1 m distance using 3D LiDAR Model-B, (**c**) map of ball at 30 cm height and 2 m distance using 3D LiDAR Model-B, (**d**) map of ball at 45 cm height and 3 m distance using 3D LiDAR Model-B, (**e**) map of ball at 1 m distance without elevation using 3D LiDAR Model-A, (**f**) map of ball at 15 cm height and 1 m distance using 3D LiDAR Model-A, (**g**) map of ball at 30 cm height and 2 m distance using 3D LiDAR Model-A, (**h**) map of ball at 45 cm height and 3 m distance using 3D LiDAR Model-A, (**i**) map of ball at 1 m distance without elevation using 32-channel LiDAR, (**j**) map of ball at 15 cm height and 1 m distance using 32-channel LiDAR, (**k**) map of ball at 30 cm height and 2 m distance using 32-channel LiDAR, (**l**) map of ball at 45 cm height and 3 m distance using 32-channel LiDAR. Dashed blue box (in panels (**a**–**h**)) and dashed red box (in panels (**i**–**l**)) mark the projected extent of the target’s ground-truth bounding box.

**Figure 14:**
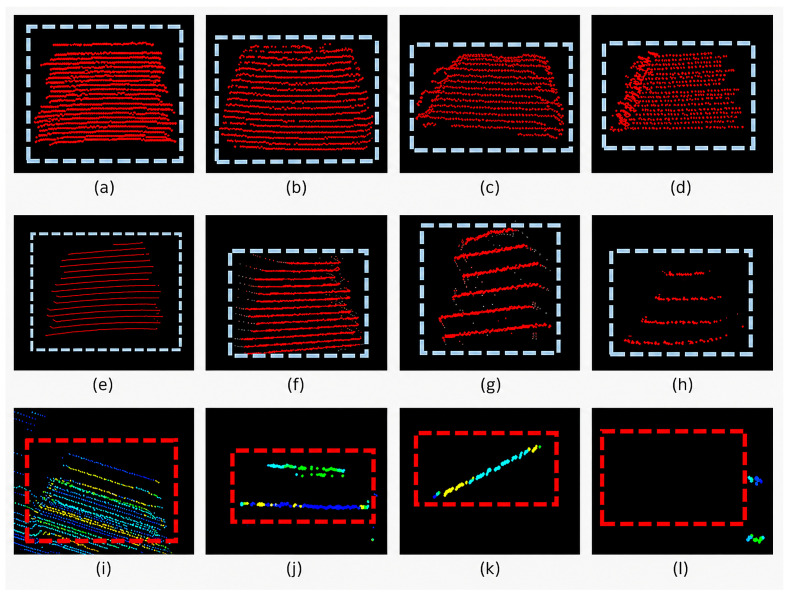
(**a**) Map of trapezoid at 1 m distance without elevation using 3D LiDAR Model-B, (**b**) map of trapezoid at 15 cm height and 1 m distance using 3D LiDAR Model-B, (**c**) map of trapezoid at 30 cm height and 2 m distance using 3D LiDAR Model-B, (**d**) map of trapezoid at 45 cm height and 3 m distance using 3D LiDAR Model-B, (**e**) map of trapezoid at 1 m distance without elevation using 3D LiDAR Model-A, (**f**) map of trapezoid at 15 cm height and 1 m distance using 3D LiDAR Model-A, (**g**) map of trapezoid at 30 cm height and 2 m distance using 3D LiDAR Model-A, (**h**) map of trapezoid at 45 cm height and 3 m distance using 3D LiDAR Model-A, (**i**) map of trapezoid at 1 m distance without elevation using 32-channel LiDAR, (**j**) map of trapezoid at 15 cm height and 1 m distance using 32-channel LiDAR, (**k**) map of trapezoid at 30 cm height and 2 m distance using 32-channel LiDAR, (**l**) map of trapezoid at 45 cm height and 3 m distance using 32-channel LiDAR. Dashed blue box (in panels (**a**–**h**)) and dashed red box (in panels (**i**–**l**)) mark the projected extent of the target’s ground-truth bounding box.

**Figure 15:**
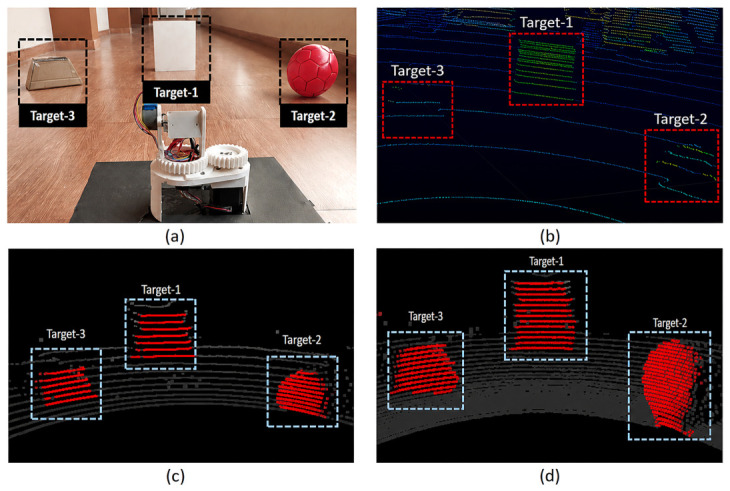
(**a**) Real-time experiment setup of scenario-1 (setup-2); (**b**) map of scenario-1 obtained using 32-channel commercial LiDAR; (**c**) map of scenario-1 obtained with the same parameters as those used for the commercial LiDAR; (**d**) map of scenario-1 obtained after customizing the parameter of our proposed 3D CS LiDAR. Dashed blue box marks the projected extent of each target’s ground-truth bounding box in the point cloud panels.

**Figure 16:**
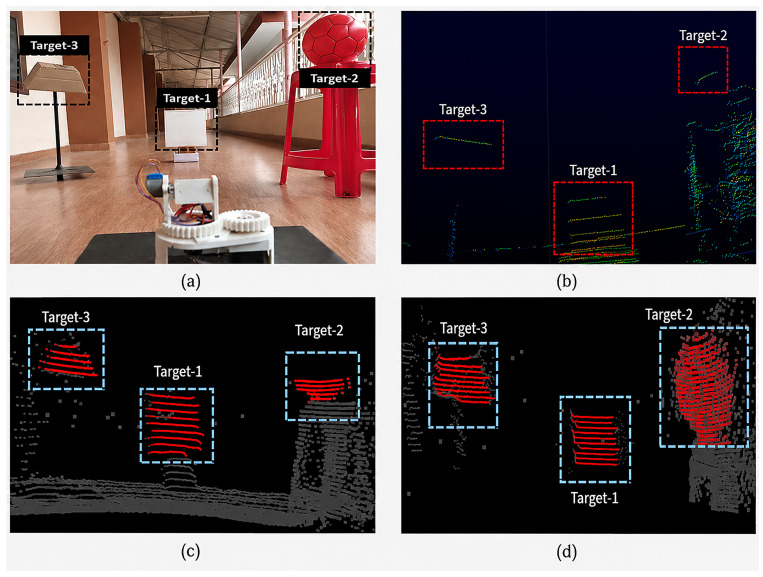
(**a**) Real-time experiment setup of scenario-2 (setup-2); (**b**) map of scenario-2 obtained using 32-channel commercial LiDAR; (**c**) map of scenario-2 obtained with the same parameters as those used for the commercial LiDAR; (**d**) map of scenario-2 obtained after customizing the parameter of our proposed 3D CS LiDAR. Dashed blue box marks the projected extent of each target’s ground-truth bounding box in the point cloud panels.

**Figure 17:**
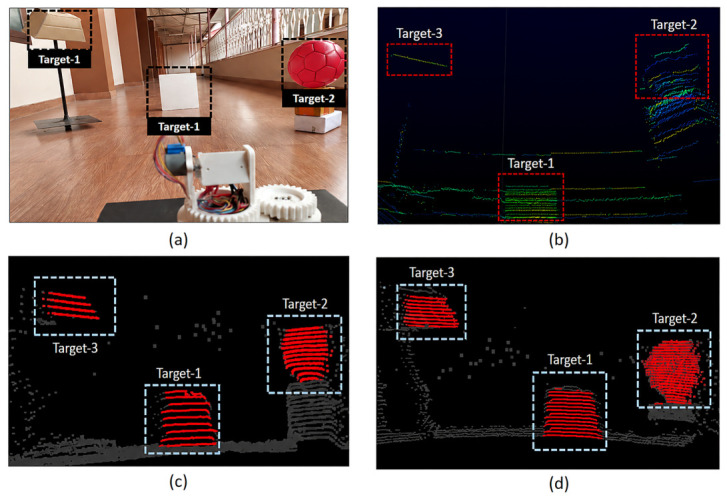
(**a**) shows real-time experiment setup of scenario-3 (setup-2), (**b**) shows the map of scenario-3, obtained using 32-channel commercial LiDAR, (**c**) shows the map of scenario-3, obtained with parameters same as that of the commercial LiDAR, (**d**) shows the map of scenario-3, obtained after customizing the parameter of our proposed 3D CS LiDAR. Dashed blue box marks the projected extent of each target’s ground-truth bounding box in the point cloud panels.

*Coordinate frame of the point cloud plots.* All point cloud visualisations in Figure 12, Figure 13, Figure 14, Figure 15, Figure 16 and Figure 17 are rendered in RViz using the LiDAR body frame defined in Figure 2: the *X* axis points forward (target-facing) along the ToF beam at azimuth α=0, the *Y* axis is horizontal and perpendicular to *X*, and the *Z* axis is vertical (elevation). Each subplot is a front-facing XZ view of the corresponding scene. The dashed blue box in the Model-A and Model-B panels, and the dashed red box in the 32-channel panels, marks the projected extent of the target’s ground-truth bounding box in the same frame; on-target points are those falling inside this box. For all subplots the visualisation scale is preserved so that layer densities can be compared visually across panels.

#### 4.1.1. Scenario-1 Plank

When the plank is placed on the ground plane parallel to the LiDAR (Figure 12a,e,i), all three sensors return usable representations of the target. The commercial 32-channel LiDAR, however, exhibits a characteristic non-uniformity: the bottom of the plank is densely packed with returns while the top is sparse. This reflects the standard scan geometry of a multi-channel LiDAR, in which the beams are concentrated near the sensor’s optical centreline. The pattern is favourable when the target lies on the same plane as the LiDAR but becomes limiting when the target is elevated: in Figure 12j–l, the plank is represented by fewer than three scan lines, and at the 45 cm elevation of Figure 12l only a single laser layer intersects the target. Model-A and Model-B of the proposed system continue to produce dense on-target representations under the same elevated conditions (Figure 12d,h). Layer density decreases with increasing distance, as expected from the constant angular resolution of both sensors, and this drop is visible in the Model-A results (Figure 12f–h). Model-B, whose finer vertical step lifts the layer count ceiling well above the $1^{\circ }$ resolution limit of Model-A, produces high-density representations in Figure 12a–d. In these tests the FoV of the proposed system was typically restricted to $95^{\circ }$–$120^{\circ }$ around the elevation of the target, which both concentrates the scan budget on the region of interest and reduces the number of returns that could be mistaken for spurious features.

#### 4.1.2. Scenario-2 Ball

Point clouds obtained with the ball as the target are shown in Figure 13. As with the plank, layer density is high at close range and decreases with distance. When the ball is elevated to 45 cm at 3 m distance, the commercial 32-channel LiDAR returns no points on the target (Figure 13l), and even at 1 m and 2 m distance the target is represented by only one or two scan lines (Figure 13i,k)—insufficient for reliable geometric recovery. Model-A (Figure 13e–h) detects the ball in every configuration, but at $1^{\circ }$ resolution the representation is comparatively sparse. Model-B (Figure 13a–d) again produces the highest layer density, representing the ball with at least 10 scan layers in every configuration and thereby yielding the highest detection confidence of the three sensors.

#### 4.1.3. Scenario-3 Trapezoid

Point clouds obtained with the trapezoid as the target are shown in Figure 14. The trends mirror those of Scenarios 1 and 2: Model-B yields the highest-resolution representation of the trapezoid across every configuration. The 32-channel LiDAR represents the trapezoid with only one or two scan layers at the 15 cm and 30 cm elevations (Figure 14j,k) and, as with the ball, fails to return points on the target at the 45 cm elevation (Figure 14l). Model-A represents the target with four scan layers at 3 m/45 cm (Figure 14h), while Model-B represents it with more than ten (Figure 14d). This confirms that the finer vertical step of Model-B preserves detection performance at longer distances where the coarser Model-A representation begins to lose target detail.

#### 4.1.4. Quantitative Comparison for Experiment-1

To supplement the qualitative comparisons above, we report two quantitative metrics extracted directly from the recorded point cloud plots of Figure 12, Figure 13 and Figure 14:

- *Scan layers on target ($L_{t}$)*: the number of distinct horizontal scan layers containing at least one point on the target object. $L_{t}$ serves as a direct proxy for the effective detection resolution on the target: a target represented by more scan layers is one that occupies more of the sensor’s angular sampling budget and can therefore be characterised more accurately by downstream algorithms.
- *Detection outcome*: a target is labelled “detected” (Y) if $L_{t}\geq 2$ (i.e., at least two scan layers intersected the target), and “missed” (N) otherwise. The latter constitutes a false negative at the target detection level.

Table 3 reports these metrics for the most demanding Experiment-1 configuration (3 m horizontal distance, 45 cm target elevation), which is the case in which the commercial 32-channel LiDAR most clearly loses target information (as observed qualitatively in Figure 12l, Figure 13l, and Figure 14l, respectively). A full per-point range accuracy characterisation (mean absolute error in centimetres) would require access to the raw range measurements of every on-target point and is discussed in the Limitations paragraph of Section 5.

The values in Table 3 quantify the qualitative claims of Section 4.1.1, Section 4.1.2 and Section 4.1.3. At the 3 m/45 cm configuration the commercial 32-channel LiDAR represents the plank with only a single scan layer ($L_{t}=1$, below the detection threshold), and fails to return any points on the ball or the trapezoid ($L_{t}=0$), yielding a false negative rate of 3/3 at this configuration. Model-A of the proposed system detects all three targets with $L_{t}\approx 4$, and Model-B further improves the representation to $L_{t}\geq 10$ for every target—an order-of-magnitude gain in on-target layer density compared to the commercial baseline. Because Model-B evenly distributes its finite scan budget across the user-specified FoV rather than concentrating layers near the sensor’s optical centre, small or off-axis targets that fall outside the commercial LiDAR’s dense central band are still resolved with high layer density.

### 4.2. Results for Experiment-2

For Experiment-2 we compare three configurations of the sensor: (i) the commercial 32-channel LiDAR at its native settings, (ii) the proposed 3D CS LiDAR configured to match the commercial sensor’s parameters, and (iii) the 3D CS LiDAR configured with the customised parameters listed in Table 4. All results are obtained with Model-B, which was shown in Experiment-1 to be the higher-resolution prototype. For the “matched” configuration in (ii), the vertical FoV was set from $65^{\circ }$ ($-25^{\circ }$) to $105^{\circ }$ ($+15^{\circ }$) to reproduce the commercial LiDAR’s specifications, and the range was fixed at 2.5 m across all cases. The parameter sets used for the customised configurations of each scenario are those of Table 4.

#### 4.2.1. Scenario-1

Figure 15 shows the ground-plane scenario, in which all three targets lie on the floor at horizontal distances ranging from 1 m to 3 m. The commercial LiDAR identifies the plank clearly (Figure 15b) but under-samples the sphere and trapezoid: the trapezoid is represented by only two scan layers, and the sphere is partially detected by the intersection of four scan layers. When the 3D CS LiDAR is configured with the same parameters as the commercial LiDAR (Figure 15c), it produces a more even distribution of layers across the FoV, giving the sphere and trapezoid additional coverage at the cost of some coverage on the plank; the sphere, however, remains partially represented. In the customised configuration (Figure 15d, parameters as in Table 4 for Scenario-1), the FoV and layer count are directed onto the three targets specifically, and a large number of scan layers intersects each target. Every target is represented in full, and the layer count budget is not spent on scene regions outside the targets.

#### 4.2.2. Scenario-2

Figure 16 shows Scenario-2, in which the sphere and trapezoid are elevated while the plank is placed on a stand at ground level. The commercial LiDAR (Figure 16b) misses substantial portions of the sphere and trapezoid because both fall near the edge of its FoV, where beam density is lowest; the plank is represented by four to five layers. The matched-parameter 3D CS LiDAR (Figure 16c) distributes layers uniformly across the FoV and thereby improves the coverage of the sphere and trapezoid, though the sphere is still only partially represented. In the customised configuration (Figure 16d, Table 4—Scenario-2), all three targets are represented at high layer density, and the chair supporting the sphere (visible in Figure 16b,c) is excluded from the point cloud through the tighter FoV. This eliminates a source of irrelevant returns that would otherwise consume downstream processing budget.

#### 4.2.3. Scenario-3

Figure 17 shows Scenario-3, in which all three targets are placed at elevations above the ground plane. The commercial LiDAR (Figure 17b) shows the expected trend: target-1 (nearest and centred) is represented well; target-2 shows lower resolution due to its elevation; and target-3 (highest elevation) is represented by very few points. The matched-parameter 3D CS LiDAR (Figure 17c) shows a similar decline in coverage with increasing target range. The customised configuration (Figure 17d, Table 4—Scenario-3) again places layers directly onto the three targets and yields the highest-density and most uniformly represented result of the three, which supports downstream perception tasks that depend on consistent point coverage across all targets in the scene.

Although the vertical resolution achievable by Model-B is bounded at $0.18^{\circ }$ (Equation (3)), the resolutions used in Table 4 are set to intermediate values in order to balance layer density against total scan time and downstream processing cost.

#### 4.2.4. Quantitative Comparison for Experiment-2

The three scenarios in Experiment-2 were also evaluated quantitatively using the $L_{t}$ (scan layers on target) and detection metrics defined in Section 4.1.4. In addition, we report the presence of significant background clutter—specifically, whether the sensor’s returned point cloud contains an extraneous structure not corresponding to any target (e.g., the chair on which the ball was placed in Scenario-2, visible in Figure 16b,c but cropped in Figure 16d). Reduction in such clutter is one of the direct benefits of the reconfigurability feature, since a customised FoV directs the sensor’s finite scan budget toward the region of interest. Table 5 summarises the results, with $L_{t}$ values reported as the mean number of scan layers per target, extracted by direct counting from the point cloud plots of Figure 15, Figure 16 and Figure 17.

Three consistent trends are visible across Table 5. First, the customised 3D CS LiDAR configuration achieves zero missed targets in all three scenarios, while the commercial 32-channel LiDAR misses one target in Scenarios 2 and 3 (the elevated ball). This represents a measurable false negative reduction under equivalent viewing conditions. Second, the customised configuration is the only one that produces no significant background clutter—the chair supporting the ball in Scenario 2, for example, is present in the commercial LiDAR’s output (Figure 16b) and in the uncustomised 3D CS output (Figure 16c), but is cropped out of the customised output (Figure 16d). Third, the mean number of scan layers per target is at least 2–4× higher for the customised configuration than for the commercial baseline, quantifying the “high resolution” claim made qualitatively in Section 4.2.1, Section 4.2.2 and Section 4.2.3.

*Data volume implication.* The number of points produced by any pulsed-ToF LiDAR per full-scene refresh scales linearly with the product of the horizontal sample count $N_{\alpha }$, the vertical layer count *n*, and the fraction of the FoV actually swept. For the commercial 32-channel LiDAR operating at its native $40^{\circ }\times 360^{\circ }$ FoV with a typical $N_{\alpha }\approx 1800$ samples per rotation, the theoretical point budget per scan is on the order of $N_{\alpha }\cdot n=1800\times 32=57,600$ points, of which only a small subset lies on any given target. In contrast, the customised Model-B configuration of Table 4 restricts the vertical FoV to at most $30^{\circ }$ with n=40–50 layers, focused entirely on the region occupied by the targets. Under these settings the point budget on the actual region of interest is preserved or increased, while the total returned point count within the scanned FoV is smaller than that of the wide-FoV commercial sensor by roughly the ratio of vertical FoV coverage—a reduction to the order of $30^{\circ }/40^{\circ }$ times the corresponding narrowing of the horizontal FoV where the customisation is applied. The proportional reduction in downstream processing cost (per-point transformations, buffering, and RViz rendering) follows directly, since the cost of the spherical-to-Cartesian mapping of Equation (1) is per-point and therefore linear in |P|. A precise wall-clock timing and memory footprint characterisation was not performed in the current study and is targeted for the follow-up work identified in the Limitations paragraph of Section 5.

Across both experimental setups, the reconfigurable 3D CS LiDAR provides denser on-target sampling and lower false negative rates than the commercial 32-channel LiDAR in every tested configuration within the indoor 1–3 m envelope evaluated here. This supports the claim that run-time control of the sensing envelope can meaningfully improve close-range indoor perception at a small fraction of the commercial sensor’s cost, while acknowledging that a broader statistical characterisation over a larger target class and range envelope is required for generalisation. Ongoing work is directed at closing the gap in scene refresh rate, which remains the principal limitation of the mechanically actuated single-ToF architecture.

## 5. Conclusions

This paper has presented 3D CS LiDAR, a reconfigurable 3D LiDAR architecture in which the field of view, the number of output layers, and the sensing range are exposed as run-time parameters of a single-ToF, dual-actuator system. Two prototypes were implemented: Model-A, using a servo-driven vertical actuator with a 1° resolution ceiling, and Model-B, which replaces the servo with a 28BYJ-48 stepper to lower the resolution ceiling to 0.18° and thereby raise the layer count budget by more than an order of magnitude. The paper described the mechanical, electrical, and software subsystems of both prototypes, formalised the spherical-to-Cartesian coordinate transformation applied to the raw sensor triples (Equation (1)), and reported specification, cost, refresh rate, and throughput characteristics in Table 1 and Table 2.

The prototypes were evaluated against a commercial 32-channel LiDAR under two experimental setups: Setup-1 (single-target scans at horizontal distances of 1–3 m and vertical elevations of 15–45 cm) and Setup-2 (multi-target scenes with the sensor mounted on a mobile robot). Under Setup-1 at the most demanding configuration (3 m distance, 45 cm elevation), Model-B represented every target with at least 10 scan layers, while the commercial LiDAR failed to detect two of three targets. Under Setup-2 the customised Model-B configuration achieved zero missed targets in all three scenarios, while the commercial LiDAR missed one target in Scenarios 2 and 3. Comparable Model-B representations were obtained at a hardware bill-of-materials cost approximately one to two orders of magnitude below that of the commercial reference sensor. Several limitations of the current work are addressed in the future work items listed below:

1. Reducing the noise (mainly due to the fringing of light rays) from the sensor to make the output map more accurate.
2. Increasing the refreshing rate which requires a major design change.
3. Improving the range of the ToF sensor to cover a larger area.
4. Automatic reconfiguration of the FoV, layer count, and range parameters based on scene content, using either classical heuristics on a low-resolution preview scan or a learned policy trained on a corpus of (scene, optimal parameter) pairs. The current work exposes the reconfigurable parameters as a run-time API; this future item closes the loop by having the perception stack select the parameters itself.
5. Outdoor characterisation of the sensor under uncontrolled illumination, humidity, and airborne particulate loading, and quantification of the range-precision degradation that these environmental variables induce on the 940 nm TF Luna module. This would extend the sensor from indoor use toward outdoor autonomous-vehicle and drone deployments.
6. Integration of a dedicated point cloud denoising and restoration stage between the ROS-python coordinate-transformation node and the visualisation output. Candidate methods from the LiDAR-specific literature include classical statistical outlier removal (SOR), radius-based outlier removal, bilateral filtering on range images, guided-image filtering for depth data, and learned point cloud denoisers such as ScoreDenoise and PointCleanNet. Analogous restoration frameworks developed for degraded 2D imagery in adjacent modalities—for example, diffusion-based [32] and Bayesian variational [33] formulations for image dehazing—may also provide methodological inspiration for point cloud restoration once the mapping between 2D image-space degradation and 3D point cloud degradation is established. This stage would directly address the fringing-noise limitation of Item 1 and is expected to reduce false feature detections in cluttered environments.
7. Wall-clock timing and memory footprint benchmarking of the customised versus uncustomised sensor configurations against the same downstream perception pipeline, to complement the data volume argument of Section 4.2.4 with directly measured processing cost figures.

*Limitations.* The quantitative results reported in this study are restricted to two figure-derivable metrics: the number of scan layers on target ($L_{t}$) and the target-level detection outcome (Y/N, with threshold $L_{t}\geq 2$). A full quantitative characterisation of the sensor—including per-point range accuracy statistics, precise on-target point counts, and a numerical false positive ratio computed against ground-truth bounding boxes—requires access to the raw range measurements of every returned point. Such measurements were not retained from the initial evaluation reported here and are targeted for a follow-up study using an instrumented indoor test range. The evaluation was performed on three geometric primitives (plank, ball, trapezoid) at horizontal distances of 1–3 m; extension to a wider class of real-world targets and to longer ranges (bounded by the TF Luna’s 8 m rated range) is left to future work. Finally, the effective full-scene refresh rate of the current mechanically actuated design is substantially lower than that of a comparable multi-channel commercial LiDAR; increasing the refresh rate is listed as Item 2 in the future work list above.

## Figures and Tables

**Table 1:** Technical specifications of the two proposed 3D CS LiDAR models and the commercial 32-channel reference LiDAR.

Parameter	Model-A	Model-B	Commercial 32-Channel
ToF ranger	TF Luna	TF Luna	Multi-channel array
Laser wavelength (nm)	940	940	905 (typ.)
Ranging principle	Pulsed ToF	Pulsed ToF	Pulsed ToF
Nominal range (m)	0.2–8	0.2–8	0.5–100
Range precision (cm)	±2 (≤3 m); ±6 (3–8 m)	±2 (≤3 m); ±6 (3–8 m)	±3 (typ.)
ToF FoV (single beam)	2°	2°	0.16° (per beam)
Azimuthal actuator	NEMA-17 stepper	NEMA-17 stepper	DC brushless spindle
Elevation actuator	Towerpro MG995 servo	28BYJ-48 stepper	(fixed beam array)
Elevation step (min.)	1°	0.18°	(fixed, 40° span)
Vertical FoV	20°–180° (user-set)	20°–180° (user-set)	40° ($-25^{\circ }$ to $+15^{\circ }$)
Horizontal FoV	0°–360° (user-set)	0°–360° (user-set)	360° (fixed)
Number of layers	up to 50 (per Equation (2))	up to 277 (per Equation (3))	32 (fixed)
Run-time reconfigurable	Yes	Yes	No
Microcontroller/SBC	Arduino UNO	Arduino UNO	Vendor firmware
Slip ring	6-wire	12-wire	N/A
Motor driver	TB6600 (stepper), direct PWM (servo)	TB6600 (stepper), ULN2003 (stepper)	N/A

**Table 2:** Indicative component-level cost of the 3D CS LiDAR prototypes (USD). Prices are typical list prices of the individual off-the-shelf modules at the time of build (2023–2024) and exclude 3D-printed mechanical parts (in-house), shipping, and taxes. The commercial 32-channel LiDAR cost is a typical list price range for entry- to mid-tier devices.

Component	Model-A (USD)	Model-B (USD)
TF Luna ToF ranger	∼22	∼22
NEMA-17 stepper motor	∼15	∼15
Vertical actuator	Towerpro MG995 servo, ∼5	28BYJ-48 stepper, ∼3
TB6600 stepper driver	∼10	∼10
ULN2003 driver	–	∼2
Arduino UNO	∼25	∼25
Slip ring	6-wire, ∼8	12-wire, ∼15
Structural/mechanical hardware (screws, brackets, wiring)	∼15	∼15
Prototype total (excl. 3D print)	∼100	∼107
Commercial 32-channel LiDAR (list price, ref.)	∼4000–8000	

**Table 3:** Quantitative comparison of Model-A, Model-B, and the commercial 32-channel LiDAR at the most demanding Experiment-1 configuration (3 m horizontal distance, 45 cm target elevation). $L_{t}$: number of scan layers containing on-target points; Det.: detected (Y/N, threshold $L_{t}\geq 2$). Values are extracted by direct counting of scan layers in the point cloud plots of Figure 12, Figure 13 and Figure 14.

Target	Sensor	$L_{t}$	Det.
	Commercial 32-ch	1	N
Plank (35×35 cm)	3D CS LiDAR Model-A	∼4	Y
	3D CS LiDAR Model-B	∼15	Y
	Commercial 32-ch	0	N
Ball (⌀22 cm)	3D CS LiDAR Model-A	∼4	Y
	3D CS LiDAR Model-B	≥10	Y
	Commercial 32-ch	0	N
Trapezoid (18/29/14 cm)	3D CS LiDAR Model-A	4	Y
	3D CS LiDAR Model-B	≥12	Y

**Table 4:** Customized parameters considered for conducting Test Setup-2.

Scenario Number	Minimum Angle	Maximum Angle	Number of Output Layers	Vertical Resolution (°)
1	60°	90°	40	0.75
2	90°	120°	50	0.60
3	75°	105°	40	0.75

**Table 5:** Quantitative comparison for Experiment-2 across the three scenarios. Values are aggregated over the three targets (plank, ball, trapezoid) per scenario. $L_{t}$: mean number of scan layers containing on-target points across the three targets; Clutter: visible extraneous background structure in the point cloud (Y/N); Miss: number of targets (out of 3) with $L_{t}<2$ (false negatives). Values extracted by direct counting from Figure 15, Figure 16 and Figure 17.

Scenario	Sensor/Configuration	$L_{t}$ (Mean)	Clutter	Miss/3
	Commercial 32-ch	∼3	Y	0
1 (ground)	3D CS LiDAR (same params as 32-ch)	∼5	Y	0
	3D CS LiDAR (customised, Table 4)	≥15	N	0
	Commercial 32-ch	∼2	Y	1
2 (mixed)	3D CS LiDAR (same params as 32-ch)	∼5	Y	0
	3D CS LiDAR (customised, Table 4)	≥12	N	0
	Commercial 32-ch	∼3	Y	1
3 (elevated)	3D CS LiDAR (same params as 32-ch)	∼6	Y	0
	3D CS LiDAR (customised, Table 4)	≥12	N	0

## Data Availability

The point-cloud datasets generated during the current study are available from the corresponding author on reasonable request.

## Abbreviations

The following abbreviations are used in this manuscript:

3D CS LiDAR	3D Customizable LiDAR
FoV	Field of View
